# SPEECH THERAPY INTERVENTION IN MORBIDLY OBESE UNDERGOING FOBI-CAPELL GASTROPLASTY METHOD

**DOI:** 10.1590/0102-6720201600010011

**Published:** 2016

**Authors:** Rosa de Fátima Marques GONÇALVES, Ethel ZIMBERG

**Affiliations:** ABC Medical School, Santo André, SP, Brazil

**Keywords:** Gastroplasty, Morbid obesity, Mastication, Quality of life, Speech therapy

## Abstract

***Background*:**

The rehabilitation of complications related to oral feeding, resulting from gastroplasty is the competence of the speech therapist, to intervene in mastication and swallowing functions, aiming at quality of life.

***Aim*:**

Check in the postoperative period the efficiency of stimulation, independent judges in readiness for re-introduction of solid food in morbidly obese undergoing gastroplasty.

***Method*:**

Cross-sectional study of descriptive and quantitative evaluated mastication and quality of life of 70 morbidly obese patients undergoing gastroplasty, and a group of 35 obese suffered speech therapy.

***Results*:**

In the evaluation of mastication for group 1 (pre and post speech therapy), the results show that, except for the lack of chewing, the other variables, such as food court, type of mastication, mastication rhythm, jaw movements, bolus size, excessive mastication and fluid intake, demonstrate statistical insignificance. In evaluating the quality of life when compared groups 1 and 2, the results from the questionnaire on quality of life in dysphagia (SWAL-QoL - Quality of Life in Swallowing) total and 11 domains assessed in the questionnaire, were statistically significant. With these results, the group 2 presented unfavorable conditions for quality of life *.*

***Conclusion*:**

The stimulation protocol, independent judges in readiness for re-introduction of solid food of these patients in the postoperative period, applied in these conditions of the study, was not the distinguishing factor of the rehabilitation process for the observed period.

## Abstract (pt)

***Racional*:**

A reabilitação das complicações relacionadas à fase oral da alimentação decorrentes da gastroplastia é de competência do fonoaudiólogo para intervir nas funções de mastigação e deglutição, objetivando a qualidade de vida.

***Objetivo*:**

Verificar a eficiência da estimulação da motricidade orofacial na prontidão para reintrodução da alimentação sólida em obesos mórbidos submetidos à gastroplastia no período pós-operatório.

***Método*:**

Estudo do tipo transversal de caráter descritivo e quantitativo avaliou mastigação e qualidade de vida de 70 obesos mórbidos submetidos à gastroplastia, sendo que um grupo de 35 obesos sofreu intervenção fonoaudiológica.

***Resultados*:**

Na avaliação da mastigação para o grupo 1 (pré e pós-intervenção fonoaudiológica), os resultados obtidos mostram que, à exceção da escassez de mastigação, as demais variáveis, como corte do alimento, tipo de mastigação, ritmo de mastigação, movimentos de mandíbula, tamanho do bolo alimentar, excesso de mastigação e ingestão de líquido, não demonstram significância estatística. Na avaliação da qualidade de vida, quando comparados os grupos 1 e 2, os resultados obtidos no questionário de qualidade de vida em disfagia (SWAL-QoL - Quality of Life in Swallowing) total e nos 11 domínios avaliados no questionário, demonstraram significância estatística. Com esses resultados, o grupo 2 apresentou condições desfavoráveis em relação à qualidade de vida.

***Conclusão*:**

O protocolo de estimulação da motricidade orofacial na prontidão para reintrodução da alimentação sólida destes doentes no período pós-operatório não foi o fator diferencial do processo de reabilitação para o período observado.

## INTRODUCTION

Obesity is a chronic disease of metabolic and/or gene origin associated with excess body fat, which can be associated with many comorbidities^8^, and is a public health problem. Morbid obesity, which comprises BMI above 40 kg/m^2^, frequently is associated with diabetes and hypertension^8^. In Brazil, obesity is rapidly growing and has a great impact on public health and costs, increasing the last six years from 11.4% to 15.8% in general population^3^.

The therapy for obesity is complex. In addition to clinical treatment, surgical therapies are carried out, among them the gastroplasty. It has various techniques, one of them is the Fobi-Capella, widely used and considered the gold standard in bariatric surgery, due to its high level of efficiency and low morbimortality^1^.

Is of great importance the patient be monitored by multidisciplinary team, since it enables better surgical outcomes in long follow-up^11^. Among the professionals, is important the speech therapist.

There is also the influence of environmental factors on the onset of obesity; therefore, only the consumption of foods with high energy, probably, can not explain the increase in overweight and obesity rates in Brazil and worldwide. The factor that must be taken into consideration is chewing because, when classified as normal (with orofacial motor integrity), is the best appetite moderator, helping digestion^5^. Therefore, is important the presence of the speech therapist in the multidisciplinary team to evaluate and, if necessary, carry out early intervention even in the preoperative period of gastroplasty.

The main study in the area, made by the group of these authors traced the chewing profile of morbidly obese patients undergoing gastroplasty, and found that obese individuals have their own characteristics, with significant changes as compared to healthy, being characterized by: integrity on form and tongue function, cheeks and jaw, no food cut, fast chewing rhythm, vertical jaw movements, large bolus and shortage of mastication^5^.

As new area for speech therapist, the rehabilitation of the morbidly obese, aiming the stimulation of orofacial motor, does not have studies to prove its effectiveness.

After tracing the chewing profile of morbidly obese undergoing gastroplasty, this study aimed to verify the stimulation efficiency of orofacial motor skills in readiness for re-introduction of solid food in these patients in the postoperative period.

## METHOD

This is a descriptive and quantitative cross-sectional study. After registration in Brazil Platform, was first submitted to the evaluation of the Instituto Evandro Chagas Ethics Committee (Bethlehem, PA) and approved under number 0035/2011. Later, it was sent to the Chief of Bariatric Surgery of the Ophir Loyola Hospital (Bethlehem, PA) Authorization to Survey n^o^. 93/2011. All participants signed a consent form, which ensured the anonymity and rights of voluntary participation in the research.

**FIGURE 1:**
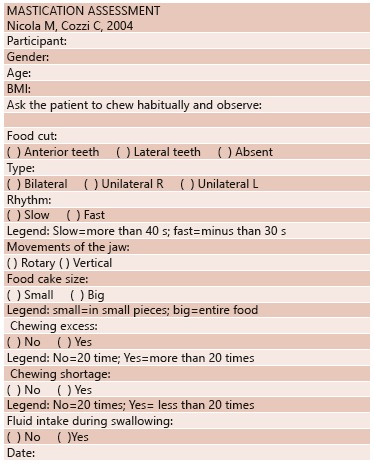
- Chew evaluation form

Postoperative period of transition from liquid to soft diet (in the range of approximately four to five weeks from operation) speech therapist applied in all participants Nicola M & Cozzi C^10^protocol for evaluation of chewing (Figure 1), making use of cheese bread, because it does not cause atypical mastication, easy to purchase, good comparison and acceptance, and that mainly favors the view during the function, since it facilitates the rotational movements during chewing.

The patient was asked to chew in usual manner and usual chewing variables, classified as food cut, type, rhythm, jaw movements, bolus size, excessive chewing, shortage of chewing and fluid intake during swallowing.

Despite the possibility of direct interference in the results, teething conditions, the use of medications, prostheses and the adjustments were not taken into account when comparing the groups, since these participants were in similar conditions, only justifying different eating habits.

For patients belonging to the group 1, one Speech Therapy Intervention Protocol containing exercise of easy access to tonus and tongue, lips and cheeks mobility was presented. After the completion and speech training, the participant was instructed to perform the exercises at home three times a day for a period of one uninterrupted month. On the back cover of this protocol was a stimulation Report Performed in which he/she marked the number of times performed the exercises series every day.

Following the protocol rules, nutrition team who attended these patients and directed them to return for reevaluation and introduction of solid consistency one month after, was asked for both groups to return to speech revaluation and application of Quality of Life Questionnaire Swallowtail-Qol. For group 1, was asked to deliver the Stimulations Performed Report already finishhed.

All participants were entered in the quality of life protocol, which used the SWAL-QoL questionnaire (Quality of Life in Swallowing - Quality of Life Questionnaire in dysphagia), already validated for other conditions and translated into Portuguese of SWAL-QoL scale. This questionnaire consists of 44 items in 11 fields that analyze: swallowing as a burden, desire to eat, time to eat, frequency of symptoms, food selection, communication, fear of eating, mental health, social functioning, sleep and fatigue. The score ranges from 0 to 100, and the lower scores represent worse quality of life in relation to dysphagia^7^.

### Participants

### Selection criteria

Were included patients diagnosed with morbid obesity undergoing gastroplasty (Fobi-Capella technique), both genders and adult age group.

Exclusion criteria were patients who had previously undergone speech therapy and having associated neurological disease (medical diagnosis).

### Statistical analysis

For statistical tests, it was adopted a significance level of 5% (p<0.05). The homogeneity test evaluated if the data associated with the categories of the variables were homogeneous or similar in the various classes or sub-categories defined by the other classification. The Mann-Whitney test evaluated the null hypothesis that the medians of two populations would be identical. The database was stored in Microsoft Excel Epi Info(r) program and statistical analysis was performed using SPSS 20.0 software.

## RESULTS

The sample consisted of 70 patients. Thirty-five were inserted in group 1 (five men and 30 women, aged 26-49 years) who received speech therapy and 35 obese inserted in group 2 (three men and 32 women, aged 25-44 years, Table 1) without speech therapy

**TABLE 1:** - Characterization of groups 1 and 2 according to gender and age

**Variables**	**Group 1 n (%)**	**Group 2 n (%)**
Gender Male Female Age (mean)	5 (14.3%) 30 (85.7%) 34.2	3 (8.6%) 32 (91.4%) 33.57
Total	35 (100%)	35 (100%)

Group 1 evaluation of mastication (pre- and post-speech intervention) on chewing cheese bread, the results showed that, except for the lack of chewing (p=0.042), the other variables, as cut food (p=0.090), type of chewing (p=0.052), chewing rhythm (p=0.097), jaw movements (p= 0.144), bolus size (p=0.144), chewing excess (p=0.087) and fluid intake (p=0.052) did not show statistical significance. Based on these results, the group 1 showed no functional difference.

In the evaluation of mastication in group 2 (pre- and post-period of one month without speech therapy) consisting of chewing the cheese bread as usual, the results showed that the variables: food court (p=0.093), type chewing (p=0.087), chewing rhythm (p=0.176), jaw movements (p=0.196), bolus size (p=0.167), excess chewing (p=0.226), chewing shortage (p=0.148) and fluid intake (p=0.168) did not show statistical significance. With these results, the group 2 showed no functional difference (Table 2).

**TABLE 2:** - Chewing evaluation

**Variables**	**1st Evaluation Group 1 n (%)**	**2nd Evaluation Group 1 n (%)**	**p**	**1st Evaluation Group 2 n (%)**	**2nd Evaluation Group 2 n (%)**	**p**
Food cut Anterior teeth Lateral teeth Absence	20 (57.1%) 3 (08.6%) 12 (34.3%)	20 (57.1%) 5 (14.3%) 10 (28.6%)	0.090	21 (60.0%) 20 (57.1%) 3 (08.6%)	22 (62.9%) 2 (05.7%) 11 (31.4%)	0.093
Type Bilateral Unilateral R Unilateral L Absence Chewing rhythm Slow Fast	18 (51.4%) 8 (22.9%) 7 (20.0%) 2 (05.7%) 13 (37.1%) 22 (62.9%)	22 (62.9%) 7 (20.0%) 6 (17.1%) 0 (00.0%) 18 (51.4%) 17 (48.6%)	0.052 0.097	19 (54.3%) 9 (25.7%) 6 (17.1%) 1 (02.9%) 13 (37.1%) 22 (62.9%)	17 (48.6%) 9 (25.7%) 7 (20.0%) 2 (05.7%) 12 (34.3%) 23 (65.7%)	0.087 0.176
Jaw movements Rotatory Vertical	15 (42.9%) 20 (57.1%)	18 (51.4%) 17 (48.6%)	0.144	15 (42.9%) 20 (57.1%)	14 (40.0%) 21 (60.0%)	0.196
Bolus size Small Big	16 (45.7%) 19 (54.3%)	19 (54.3%) 16 (45.7%)	0.144	16 (45.7%) 19 (54.3%)	14 (40.0%) 21 (60.0%)	0.167
Chewing excess No Yes	28 (80.0%) 7 (20.0%)	23 (65.7%) 12 (34.3%)	0.087	29 (82.9%) 6 (17.1%)	30 (85.7%) 5 (14.3%)	0.226
Chewing shortage No Yes	15 (42.9%) 20 (57.1%)	22 (62.9%) 13 (37.1%)	0.042	16 (45.7%) 19 (54.3%)	13 (37.1%) 22 (62.9%)	0.148
Liquid intake No Yes	19 (54.3%) 16 (45.7%)	26 (74.3%) 9 (25.7%)	0.052	20 (57.1%) 15 (42.9%)	19 (54.3%) 16 (45.7%)	0.168

Tasks performed by the Speech Therapy Intervention Protocol, held only by the group 1 consisted of at least 89% minimum percentage, displayed only by one patient, and a maximum of 100% percentage, presented by 24. With these results, group 1 presented excelente participation (Table 3).

**TABLE 3:** - Percentage of tasks execution by participants

**Execution percentage (%)**	**n**
89 92 94 96 97 98 100	1 1 2 2 3 2 24
Total	35

In evaluating the quality of life when compared to groups 1 and 2, results in total SWAL-QOL (p=0.00) and feeding areas as a burden (p=0.001), desire to eat (p=0.002), feeding duration (p=0.026), frequency of symptoms (p=0.000), selection of food (p=0.003), communications (p= 0.002), fear of feeding (p=0.000), mental health (p=0.001), social (p=0.002), sleep (p=0.003), fatigue (p=0.002) showed statistical significance. With these results, group 2 had unfavorable conditions with regard to quality of life (Table 4).

**TABLE 4:** - SWAL-QOL evaluation

	**Group 1**	**Group 2**	**Test**	**Significance**	
**SWAL-QOL total**	**66.81 (3.54)**	**62.48 (1.94)**	**Mann- Whitney U**	**0.000**	
Domains	Feeding as a burden	74.00 (9.46)	68.57 (9.74)		0.001
	Desire for food	55.81 (8.26)	55.24 (7.68)		0.002
	Feeding duration	24.29 (5.58)	26.86 (8.32)		0.026
	Frequency of symptoms	85.51 (5.63)	74.04 (3.58)		0.000
	Selection of food	61.43 (17.17)	60.57 (21.69)		0.003
	Communication	65.71 (16.50)	64.29 (18.99)		0.002
	Fear of food	48.86 (12.43)	45.00 (7.57)		0.000
	Mental health	77.29 (12.21)	76.43 (9.44)		0.001
	Social	48.80 (10.97)	48.34 (9.24)		0.002
	Sleep	71.43 (13.32)	72.00 (14.51)		0.003
	Fatigue	63.05 (8.45)	63.62 (10.40)		0.002

## DISCUSSION

Despite the potential complications associated with the gastroplasty (related to oral feeding) are often cited in the literature, there are no studies addressing the speech therapy in this group.

The interest in the intervention study started knowing the chewing profile in morbidly obese present own characteristics, with significant changes, such as integrity of form and language function, cheeks and jaw; no food cut; masticatory fast pace; vertical jaw movements; size large bolus and shortage of chewing.

Currently, the realization of gastroplasty has higher incidence in women^6^^-^ 9. In agreement with the findings in this study, which included 85.7% of obese women in group 1 and 91.4% in group 2, it was possible to explain the fact based in cultural parameter of thinness, as the ideal body, associated with femininity^9^ .

Patients who undergo this type of operation have a mean age of 36.07 years (17-66)^4^. This study is in agreement with the literature, presenting the group 1 average of 34.2 years (26-49), and in group 2 of 33.5 years (25-44).

In the evaluation of mastication, there was no statistically significant difference in group 1 after one month of application of the Speech Therapy Intervention Protocol, as well as in group 2, which was not implemented the protocol. Taking into consideration that chewing profile of morbidly obese has cut absence of food, fast chewing rhythm, vertical jaw movements, large bolus size and shortage of mastication^5^, it is noteworthy that in the period of one month after the release of pasty consistency (currently accounting for two months of operation) was not yet possible to see change in the chewing pattern, since, as a limitation of the study, there was the need for periodic evaluation protocol for the effectiveness of monitoring.

However, it raises the hypothesis that, if the revaluation of mastication was performed after the reintroduction of solid diet, possibly would get different results, since the re-established function could enhance the results.

The revaluation time decision was limited to the routine care that these patients developed more specifically hospital nutritional dynamics, which evolved the diet consistencies (liquid/pasty/solid) every month. Unfortunately, after the release of solid diet, the follow-up was unable due to discharge of the patient by the medical staff. It is noteworthy that the vast majority of patients were from the interior of the country, making difficult access to them; beyond that, the public hospital that hosted the research, has no infrastructure for weekly follow-up outpatient care.

The body fat is related to the mastication; therefore, the masticatory efficiency and age can influence the IMC^12^. Obese individuals, depending on the facial fat, possibly, have decreased tone of the lips and tongue, and may change the chewing performance, quality and the bolus deglution^2^.

In the literature there isn't specific protocol for rehabilitation of the morbidly obese.

Therefore, this study designed a Speech Therapy Intervention Protocol, which contained isotonic and isometric exercises of the tongue, lips and cheeks. The strategy in developing the protocol was based in being easy and understandable by the participants, taken into consideration the education level of the participants, since they were monitored by public health system - Unified Health System (SUS). Therefore, were used facilitated language and low degree of difficulty.

The effectiveness of the strategy for participation and execution of tasks in the protocol has been proven in group 1, through Performed Stimulations Reports in return for reassessment of chewing. It consisted of a minimum percentage of 89%, displayed only by one patient and at most 100% percentage shown by 24.

With regard to the aspects of quality of life, it was noted that the analysis of the results between groups 1 and 2, full SWAL-QOL and all assessed areas (food as a burden, desire to eat, duration of feeding, frequency symptoms, food selection, communication, fear of eating, mental health, social, sleep and fatigue) showed that group 1 had more favorable conditions for quality of life.

With these results, it was observed that group 1 showed better quality of life compared to group 2. However, this is not reflected in the result of chewing, which may indicate the disassociation between the domains evaluated and the function of the inability on the part of these patients.

The speech therapy should be directed to each individual, not limited to the application of global stimulation protocols because, despite the morbidly obese have characteristic chewing profile and improvement in quality of life in patients who underwent intervention, protocol applied in the conditions of this study was not the distinguishing factor of the rehabilitation process for the observed period. 

## CONCLUSION

The stimulation protocol of orofacial motor skills in readiness for re-introduction of solid food of these patients in the postoperative period, applied in the conditions of the study, was not the distinguishing factor of the rehabilitation process for the observed period. 
